# Temporal Variation in Sindbis Virus Antibody Prevalence in Bird Hosts in an Endemic Area in Sweden

**DOI:** 10.1371/journal.pone.0162005

**Published:** 2016-08-31

**Authors:** Jenny Christina Hesson, Jan O. Lundström, Atalay Tok, Örjan Östman, Åke Lundkvist

**Affiliations:** 1 Zoonosis Science Center, Department of Medical Biochemistry and Microbiology (IMBIM), Uppsala University, Uppsala, Sweden; 2 Department of Epidemiology and Population Health, Institute of Infection and Global Health, University of Liverpool, Liverpool, United Kingdom; 3 Swedish Biological Mosquito Control Project, Nedre Dalälvens Utvecklings AB, Gysinge, Sweden; 4 Department of Aquatic Resources, Institute of Coastal Research, Swedish University of Agricultural Sciences, Öregrund, Sweden; 5 Laboratory of Clinical Microbiology, Uppsala University Hospital, Uppsala, Sweden; University of California Davis, UNITED STATES

## Abstract

Sindbis virus (SINV) is a mosquito-borne bird virus that occasionally causes human disease in Fennoscandia, suggested to have cyclic 7-year intervals between outbreaks. Reliable data on human infections in Sweden is however lacking. Here we investigated the SINV antibody prevalence among birds in a Swedish area endemic to SINV to scrutinize if a cyclic variation in antibody prevalence is present in the natural host of SINV. Serum from birds were sampled in the summers of 2002–2004 and 2009 in the floodplains of the River Dalälven in central Sweden, with 2002 and 2009 representing hypothesized years of SINV outbreaks. A total of 963 birds from 52 species (mainly passerines) were tested for the presence of SINV antibodies using a plaque reduction neutralization test. The highest SINV antibody prevalence was found in Turdidae species, specifically Fieldfare, Redwing and Song thrush in which more than 70% of sampled individuals had antibodies to SINV in 2009. The SINV antibody prevalence significantly varied between years with 2% in 2002, 8% in 2003, 14% in 2004 and 37% in 2009. Antibodies were found equally often in hatchlings and in adults and increased from early to late in the season. Clearly, the SINV antibody prevalence was not elevated in the bird hosts in the predicted outbreak year 2002, thus solid evidence of a cyclic occurrence of SINV in Sweden is still lacking.

## Introduction

Cyclic phenomena in nature, involving parasites and their hosts are often discussed for rodent borne hantaviruses and tularaemia in Fennoscandia [1,2]. Far less investigated is the dynamics of Sindbis virus (SINV, *Togaviridae*: *Alphavirus)*, a mosquito-borne bird virus that occasionally causes disease in humans in this region. The disease goes under the name Ockelbo disease in Sweden and Pogosta disease in Finland and manifests with rash, fever and joint pains, which in some cases develop into chronic arthritis [3,4]. Based on the numbers of reported human cases in Finland, Brummer-Korvenkontio et al. [5] suggested a cyclic occurrence of SINV with peaks of infection every 7^th^ year in Fennoscandia, potentially driven by herd immunity among birds. Since then, the concept of a 7-year cycle has been widely quoted but never thoroughly investigated in neither Finland nor Sweden [4,6–15]. In Sweden, the awareness of disease caused by SINV is low and human cases are not notifiable. Human data usually involve only a couple of known cases per year (range 0–66) and peak numbers of disease have been reported during two of the expected outbreak years: 57 cases in 1988 and 46 cases 1995. However in 2013 (an intermediate year according to the hypothesis) SINV unexpectedly infected more than 50 people in a small town, far north of its normal range [16,17]. The human antibody prevalence in endemic areas in Sweden has been estimated to 3.6% [18].

Birds are the natural hosts of SINV, and five previous studies have thoroughly investigated the bird species involved in the transmission of the virus in Sweden [19–23]. Studies of SINV antibody prevalence in wild and captive birds have shown that a wide variety of species are infected by SINV in nature, and they represent all orders that have been tested (Passeriformes, Galliformes and Anseriformes) [19,20,23]. The overall SINV antibody prevalence found in the Swedish bird population measures roughly 8% [20,23], with the highest prevalence found in the passerine species Fieldfare (*Turdus pilaris*) (43.3%), Redwing (*Turdus iliacus*) (37%) and Song thrush (*Turdus philomelos*) (22.2%) [20]. Experimental SINV infection in 14 bird species (Passeriformes, Galliformes and Anseriformes) with SINV showed that all species tested produced a viremia sufficient to infect the main enzootic mosquito vector *Culex torrentium*, i.e. all reached a titer ≥ 10^3.7^ plaque forming units per millilitre (PFU/ml). Most passerine species, including Fieldfare, Redwing and Song thrush, reached viremia titers that would also be sufficient to infect the suggested mosquito bridge vector *Aedes cinerus*, i.e. a titer of >10^5^ PFU/ml. Also, the duration of viremia was long lasting (≥ 3 days) in most Passeriformes, sometimes reaching beyond the 5 days testing period [22,24,25]. Based on these studies, and the high population densities of these species in endemic areas, Passeriformes are considered the most important hosts for SINV in Sweden, with thrushes (Fieldfare, Redwing and Song thrush in particular) proposed as the main amplifying hosts [20].

This study aimed to investigate the temporal variation of SINV infections in its natural bird hosts in Sweden, in relation to the hypothesis of a cyclic occurrence of outbreaks every 7^th^ year in Fennoscandia. By investigating the prevalence of SINV neutralizing antibodies in the host population in two hypothesized outbreak years (2002 and 2009) and two intermittent years (2003 and 2004) we addressed the following main questions: (1) does the SINV neutralizing antibody prevalence vary in bird populations over time and (2) is SINV neutralizing antibody prevalence higher in bird populations during hypothesized outbreak years than during intermittent years?

## Materials and Methods

### Bird collection and sampling

Birds were caught in the River Dalälven floodplains in central Sweden during the summers of 2002 (June 6^th^ to September 8^th^), 2003 (May 29^th^ to September 7^th^), 2004 (May 13^th^ to August 20^th^) and 2009 (May 14^th^ to September 16^th^). These floodplains are within the main endemic area for SINV [18], and the virus has been isolated from mosquitoes collected in this region [26]. The collections were performed at several study sites (Fågle (60.2420° N; 16.7294° E), Gysinge (60.2913° N; 16.8690° E), Koversta (60.2936° N; 16.8319° E), Norrån (60.3064° N; 16.7738° E), Tärnsjö (60.1600° N; 16.9062° E), Valmbäcken (60.2973° N, 16.8415° E) and Österfärnebo (60.3067° N; 16.7997° E)) all located around Lake Färnebofjärden in the centre of the River Dalälven floodplains. The study sites were in alder swamps, bush-lands of wet meadows, mixed humid deciduous and coniferous forest, and areas near houses. Bird collections were performed with Japanese mist nets operated in mornings and evening, and collected birds were identified to species, sex and age [27]. Birds hatched during the current season are referred to as hatchlings and birds born any previous season are referred to as after hatching year birds. Each bird was marked with a unique coded metal leg-ring [28], and then sampled for 0.1 ml of blood from the jugular vein. The blood-samples were centrifuged within a few hours to separate serum from cells and thereafter stored at -20°C until analyzed.

### Plaque reduction neutralization test (PRNT)

The serum-samples were analysed by a SINV plaque reduction neutralization test (PRNT) using confluent Vero cells and the SINV Edsbyn 5/82 strain (passage four) as antigen. In each round of experiments a positive control (human serum from a SINV infected patient) and a negative control (uninfected rabbit serum) were included. Serum samples were diluted 1:10 to give an endpoint dilution of 1:20 when mixed with an equal volume of diluted virus. The virus was diluted to produce 80–120 plaque-forming units (PFUs) per well in a 24-well cell culture plate (Corning Costar, USA). Each serum-virus dilution was allowed to react for one hour before being seeded onto duplicate wells and overlaid by 0.5 ml per well of a mixture containing 2% agar Noble dissolved in water (Sigma-Aldrich, MO, USA), 2X Minimum Essential Medium (MEM; Life Technologies, CA, USA) and 0.04% DEAE-Dextran, and incubated at 37°C over night. On day two all wells were overlaid a second time by a mixture of the same composition with neutral red added for visualization of plaques [19]. The plates were again incubated over night before the number of plaques formed by non-neutralized virus was counted. A sample was considered positive for SINV neutralizing antibodies when the number of plaques were reduced by > 80% compared to a control containing virus only.

### Statistics

To evaluate the variation in SINV antibody prevalence we used generalised linear models with a logit-link function, using the ‘glm’-function in R3.1.1. [29]. If a bird had been infected (1) or not (0) was used as dependent variable. Taxonomic group, year, sex and age (hatchling or after hatching year) of the bird were used as category variables, and Julian day was used as a continuous variable. Type-II ANOVA-tables were calculated using the ‘car’-function in R3.1.1. [30].

### Ethics statement

The study was carried out on private ground and all landowners gave the permission to conduct the study on their respective properties. The bird catching and ring-binding was approved by the Swedish Museum of Natural History and appropriate approvals for taking blood samples from birds were obtained from the Uppsala Ethical Committee (C106/99 for 2002–2004, C129/9 for 2009).

## Results

In total, 963 birds from 52 species were captured and analyzed for SINV neutralizing antibodies using PRNT. Mainly Passeriformes were captured and analyzed (44 species, 948 individuals) but also Piciformes (4 species, 9 individuals), Acciplitriformes (1 species, 2 individuals) and Charadriiformes (3 species, 4 individuals). Within Passeriformes, species from 15 families were analyzed with Turdidae species as the most common (7 species, 383 individuals), followed by Sylviidae (12 species, 218 individuals), Paridae (6 species, 122 individuals) and Fringillidae (4 species, 74 individuals).

In total, 193 individual birds from 21 species were found with SINV neutralizing antibodies, of which all were Passeriformes except for 2 positive individuals belonging to Charadriiformes (Eurasian woodcock) and Piciformes (Lesser spotted woodpecker) (Table 1). Antibody prevalence differed between the families (χ^2^ = 19, df = 4, P<0.001), and were highest within Sylviidae (28%) where 5 of 12 investigated species had antibodies, and Turdidae (26%) where 5 of 7 investigated species had antibodies.

**Table 1:** Bird species with Sindbis virus antibodies during four years in the River Dalälven floodplains, Sweden.

Order	Family	Species	2002	2003	2004	2009	Total
Piciformes	Picidae	Lesser spotted woodpecker (*Dendrocopos minor*)	-^A^	-	1/1 (100)^B^	-	1/1 (100)
Charadriiformes	Scolopacidae	Eurasian woodcock (*Scolopax rusticola*)	-	-	1/1 (100)	-	1/1 (100)
Passeriformes	Corvidae	Eurasian jay (*Garrulus glandarius*)	-	0/1	1/1 (100)	1/1 (100)	2/3 (33)
	Laniidae	Red-backed shrike (*Lanius collurio*)	-	-	0/2	2/7 (29)	2/9(22)
	Emberizidae	Yellowhammer (*Emberiza citrinella*)	0/2	0/3	1/3 (33)	0/10	1/18 (6)
	Prunellidae	Dunnock (*Prunella modularis*)	0/7	2/12 (17)	0/9	4/15 (27)	6/43 (14)
	Fringillidae	European greenfinch (*Carduelis chloris*)	0/1	-	1/2 (50)	2/3 (67)	3/6 (50)
		Common chaffinch (*Fringilla coelebs*)	0/3	0/14	1/24 (4)	4/7 (57)	5/48 (10)
		Eurasian bullfinch (*Pyrrhula pyrrhula*)	0/2	0/2	0/4	4/5 (80)	4/13 (31)
		Total Fringillidae	0/6	0/16	2/30 (6)	10/15 (50)	12/67 (20)
	Paridae	Eurasian blue tit (*Parus caeruleus*)	-	0/1	1/9 (11)	1/8 (13)	2/18 (11)
		Great tit (*Parus major*)	1/16 (6)	1/16 (6)	2/19 (11)	6/36 (17)	10/87 (11)
		Total Paridae	1/16 (6)	1/17 (6)	3/28 (11)	7/44 (16)	12/105 (11)
	Sylviidae	Marsh warbler (*Acrocephalus palustris*)	-	-	1/1 (100)	-	1/1 (100)
		Willow warbler (*Phylloscopus trochilus*)	0/7	0/14	0/12	10/57 (18)	10/89 (11)
		Eurasian blackcap (*Sylvia atricapilla*)	1/8 (13)	3/12 (25)	7/17 (41)	8/14 (57)	19/51 (37)
		Garden warbler (*Sylvia borin*)	0/7	6/13 (46)	3/4 (75)	16/29 (55)	25/53 (47)
		Common whitethroat (*Sylvia communis*)	-	-	-	1/7 (14)	1/7 (14)
		Total Sylviidae	1/22 (5)	9/39 (23)	11/34 (30)	35/107 (33)	56/202 (28)
	Turdidae	European robin (*Erithacus rubecula*)	1/34 (3)	0/49	2/25 (8)	23/38 (61)	25/146 (17)
		Redwing (*Turdus iliacus*)	0/7	1/19 (5)	6/32 (19)	16/21 (77)	23/79 (29)
		Common blackbird (*Turdus merula*)	0/21	2/17 (12)	4/29 (14)	22/39 (56)	28/106 (26)
		Song thrush (*Turdus philomelos*)	0/6	1/7 (14)	0/7	12/17 (71)	13/37 (35)
		Fieldfare (*Turdus pilaris*)	-	0/1	-	10/12 (83)	10/13 (78)
		Total Turdidae	1/68 (1)	4/93 (4)	12/93 (13)	83/127 (65)	100/381 (26)
Total*			3/121 (2)	16/181 (9)	32/202 (16)	142/326 (44)	193/830 (23)

^A^ Number of PRNT positive individuals/number of tested individuals (percentage positive)

^B^ No individuals tested

* The table total differs slightly from the overall antibody prevalence, since 133 individuals of 31 bird species without SINV neutralising antibodies are not included in the table for clarity. These are (alphabetically ordered): 1 Barred warbler (*Sylvia nisoria*), 1 Common grasshopper warbler (*Locustella naevia*), 1 Common redstart (*Phoenicurus phoenicurus*), 5 Common reed buntings (*Emberiza schoeniclus*), 2 Common snipes (*Gallinago gallinago*), 2 Common starlings (*Sturnus vulgaris*), 6 Eurasian nuthatches (*Sitta europaea*), 2 Eurasian sparrow hawks (*Accipiter nisus*), 7 Eurasian tree creepers (*Certhia familiaris*), 2 Eurasian tree sparrows (*Passer montanus*), 9 Eurasian wrens (*Troglodytes troglodytes*), 2 Eurasian wrynecks (*Jynx torquilla*), 7 Eurasian siskins (*Carduelis spinus*), 2 European crested tits (*Parus cristatus*), 1 European green woodpecker (*Picus viridis*), 10 European pied flycatchers (*Ficedula hypoleuca*), 5 Great-spotted woodpeckers (*Dendrocopos major*), 1 Green sandpiper (*Tringa ochropus*), 5 Icterine warblers (*Hippolais icterina*), 4 Lesser whitethroats (*Sylvia curruca*), 3 Long-tailed tits (*Aegithalos caudatus*), 6 Marsh tits (*Parus palustris*), 1 Meadow pipit (*Anthus pratensis*), 1 Mistle thrush (*Turdus viscivorus*), 2 River warblers (*Locustella fluviatilis*), 2 Sedge warblers (*Acrocephalus schoenobaenus*), 12 Spotted flycatchers (*Muscicapa striata*), 20 Tree pipits (*Anthus trivialis*), 4 White wagtails (*Motacilla alba*), 6 Willow tits (*Parus montanus*), 1 Wood warbler (*Phylloscopus sibilatrix*).

**Fig 1:**
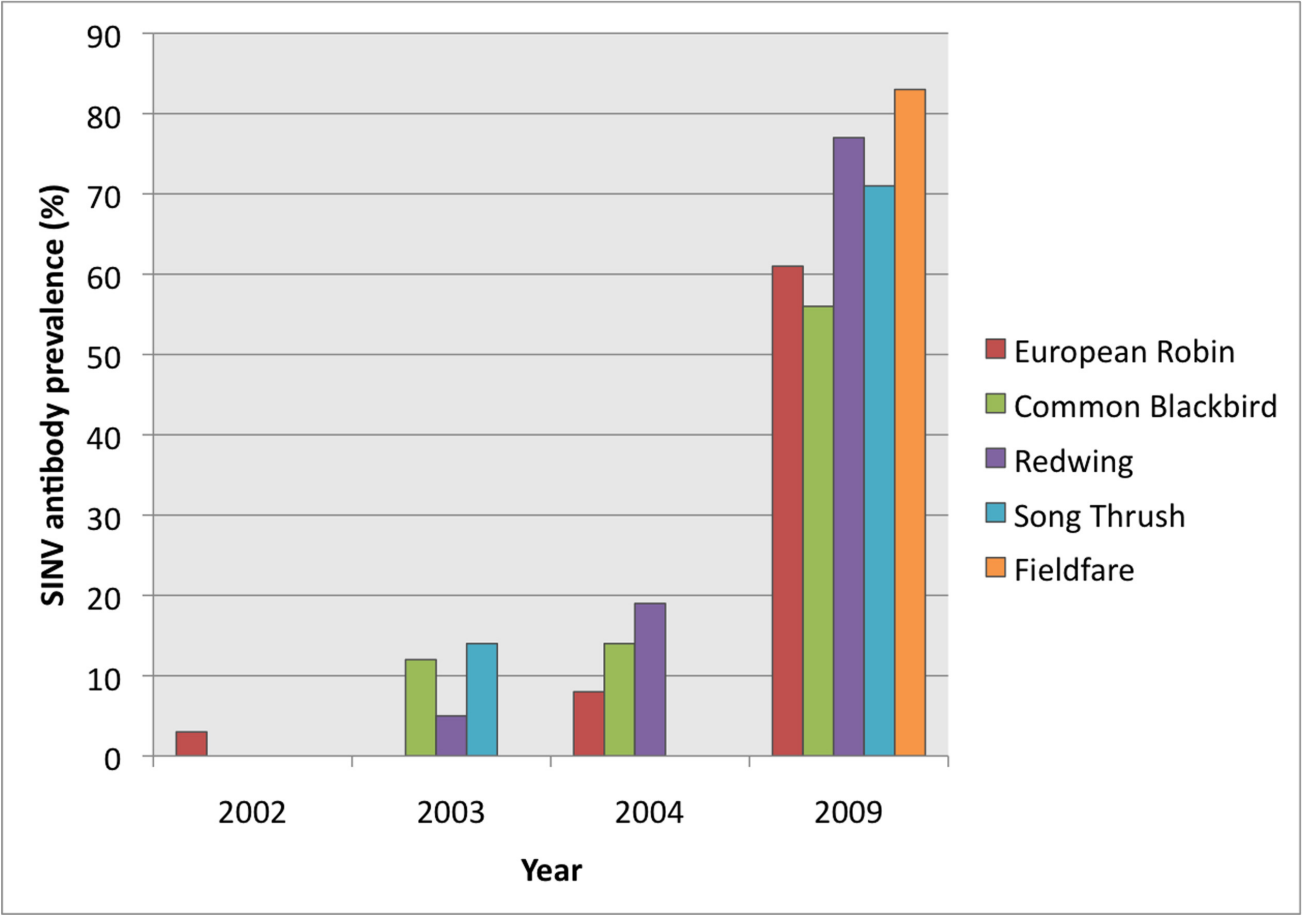
Sindbis virus neutralizing antibody prevalence in species of thrushes in the River Dalälven floodplains, Sweden.

The SINV antibody prevalence varied over years with an overall prevalence of 2% in 2002, 8% in 2003, 14% in 2004 and 37% in 2009 (χ^2^ = 132, df = 3, P<0.001). A significant yearly variation could be seen for Turdidae (χ^2^ = 115, df = 3, P<0.001) and Fringiidae (χ^2^ = 15, df = 3, P = 0.002), but not for Paridae (χ^2^ = 1.6, df = 3, P = 0.7) and Sylviidae χ^2^ = 5.1, df = 3, P = 0.2 (Table 1). Most families and individual species reached their maximum antibody prevalence in 2009, including all five Turdidae species found with antibodies against SINV (the European Blackbird, Redwing, Song thrush, European Robin and Fieldfare) (Fig 1). There was a significant difference in total SINV antibody prevalence between species within Turdidae (χ^2^ = 12, df = 6, P = 0.05), highest in the Fieldfare (78%) and lowest in the European Robin (17%) (Table 1).

Two long distance migrants were tested for antibodies against SINV; the European pied flycatcher (n = 10) and the Willow warbler (n = 89). SINV antibodies were only detected in the Willow warbler, which had a prevalence of 18% in 2009 (n = 57). Five of the positive individuals were sampled in early summer (first positive bird sampled on May 20^th^), one in the middle of summer, and four in late summer. No SINV antibodies were detected in Willow warblers collected in 2002–2004 (n = 33).

There was no effect of age on antibody prevalence, as 20% of all hatchlings (67/334) and 20% of all after hatching year birds (126/629) had antibodies against SINV. Hatchlings made up roughly one third (67/193) of all seropositive birds and the SINV antibody prevalence in hatchlings differed between years in a similar fashion as for all birds in total: 3% in 2002, 5% in 2003, 7% in 2004 and 44% in 2009 (χ^2^ = 77, df = 3, P<0.001) (Table 2).

**Table 2:** Within-season variation of Sindbis virus antibody prevalence in birds in the River Dalälven floodplains, Sweden.

	Age	2002	2003	2004	2009	Total
Early summer	All birds	0/13	4/81 (5)^A^	17/107 (16)	37/161 (23)	58/362 (16)
	Hatchlings	0/3	0/0	0/6	0/1	0/10
Mid summer	All birds	1/89 (1)	6/63 (10)	13/108 (12)	22/58 (38)	42/318 (13)
	Hatchlings	0/44	0/28	3/40 (8)	1/10 (10)	4/122 (3)
Late summer	All birds	2/38 (5)	6/62 (10)	2/19 (11)	83/164 (51)	93/283 (33)
	Hatchlings	2/22 (9)	4/47 (9)	1/13 (8)	56/120 (47)	63/202 (31)
**Total**	All birds	3/140 (2)	16/206 (7)	32/234 (14)	142/383 (37)	193/963 (20)
	Hatchlings	2/69 (3)	4/75 (5)	4/59 (7)	57/131 (44)	67/334 (20)

Sindbis virus (SINV) prevalence in early summer (May 13 –June 19), mid summer (June 20—August 10) and late summer (August 11- September 16) shown over four years and for all birds tested or hatchlings only.

^A^ Number of PRNT positive individuals/number of tested individuals (percentage positive)

**Fig 2:**
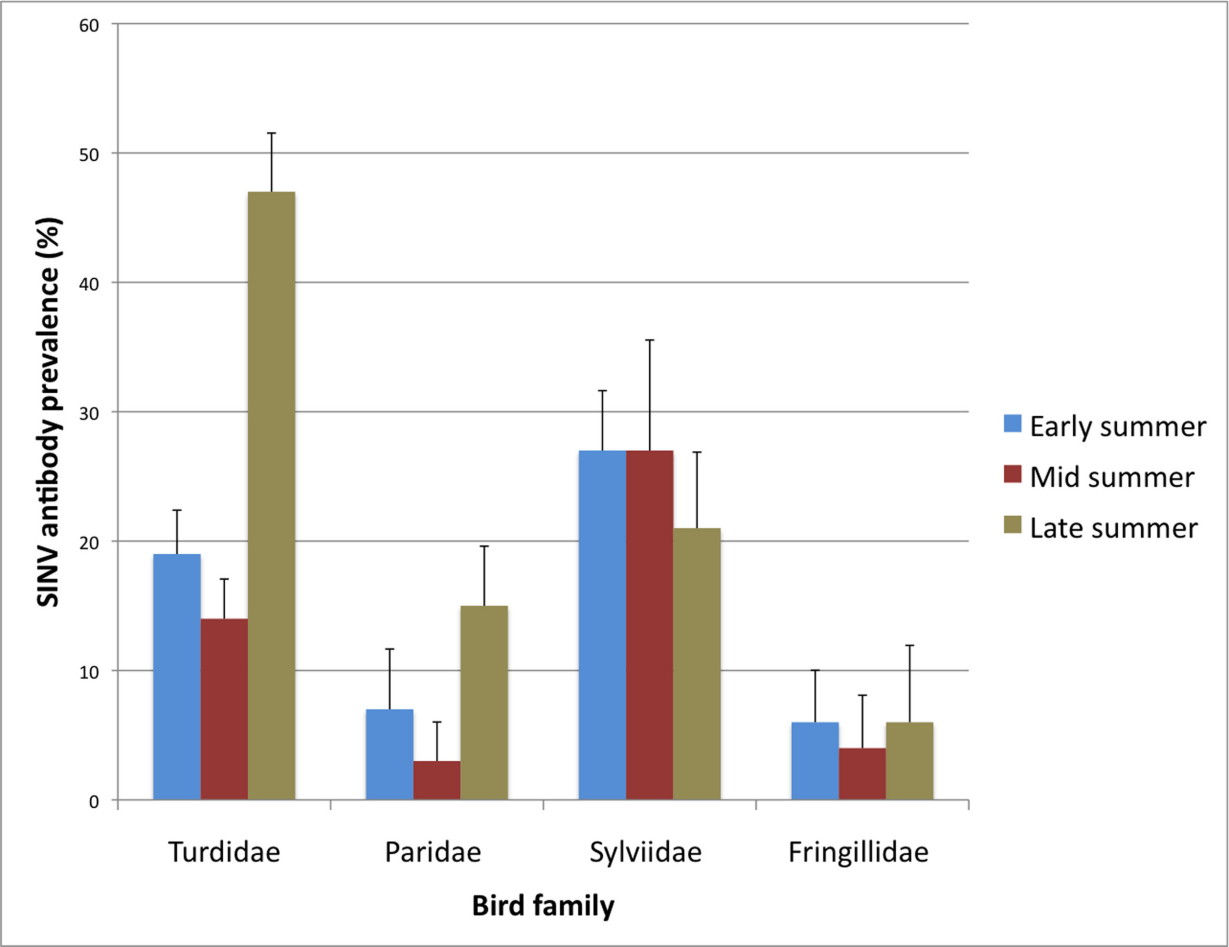
Prevalence of Sindbis virus neutralizing antibodies in four different passerine families. Error bars indicate SEM.

The earliest hatchlings with antibodies were a European robin sampled on June 21^st^ 2004, a Great tit sampled on June 24^th^ 2004 and a Song thrush sampled on June 28^th^ 2009. Earlier in the season, 58 after hatching year birds had antibodies against SINV, the earliest being two Fieldfares sampled on May 13^th^ 2004. Overall, the antibody prevalence increased between May and September (χ^2^ = 28, df = 1, P<0.001) (Table 2). This was mainly due to a seasonal increase in Turdidae (χ^2^ = 24, df = 1, P<0.001), and could not be seen for Paridae (χ^2^ = 0.2, df = 1, P = 0.6), Fringillidae (χ^2^ = 3.1, df = 1, P = 0.078) or Sylviidae (χ^2^ = 0.3, df = 1, P = 0.6) (Fig 2). This seasonal increase was also significant for hatchlings (χ^2^ = 5.8, df = 1, P = 0.016) (Table 2).

No association between SINV antibody prevalence and sex (χ^2^ = 1.5, df = 1, P = 0.2) was detected.

## Discussion

The temporal variation in the enzootic activity of SINV was investigated in a SINV endemic area in Sweden in 2002, 2003, 2004 and 2009, by measuring SINV neutralizing antibody prevalence in wild birds. Despite varying sampling success between years and species the results showed a significant variation in SINV antibody prevalence over years, however not in a cyclic 7-year fashion as suggested by an existing hypothesis [5]. According to the mentioned hypothesis, year 2002 and 2009 should have been years of high virus transmission. However, the avian antibody prevalence in 2002 was only 2%, and was exceeded by all other years investigated. Thus, potential herd immunity in the host population in the investigated region does not coincide with the suggested cycle. The SINV infection rate in enzootic vector mosquitoes collected in the same region was higher in 2002 compared to 2001 and 2003 (J.O. Lundström and J.C. Hesson unpublished data), but this appears not to have been sufficient to induce a detectable antibody prevalence response in neither the bird host or number of human cases (four were reported in 2002) [16].

Year 2009 stands out as a year with intense enzootic transmission in several aspects. The overall (and Passeriformes only) antibody prevalence in 2009 reached 37%, which can be compared to 27% (n = 51) in Passeriformes in 1988, another hypothesised outbreak year, where 57 human cases were reported in Sweden [16,23]. Earlier investigations of temporal variation in Passeriformes in adjacent areas in Sweden have measured 12.1% and 8.4% in 1990 and 1994, and 2.2% and 11.5% in 1996 and 1997 [20]. In 2009, the antibody prevalence in Fieldfare, Song thrush and Redwing all reached over 70%, which is among the highest SINV prevalence ever measured for an individual bird species. Only the 94% SINV antibody prevalence reported for the Olive thrush (*Turdus olivaceus*) in South Africa [31] is higher than the prevalence observed for *Turdus* species in Sweden. Attempts to detect and isolate virus in the enzootic mosquito vector population also indicated high SINV transmission in 2009 [26]. SINV was isolated with the highest infection rate (IR) ever recorded for the enzootic vectors that are responsible for the bird-to-bird transmission: 36 infected mosquitoes of 1000 *Cx*. *torrentium* and eight infected mosquitoes per 1000 *Culex pipiens*. Despite this, the numbers of reported human cases were only six in 2009 [16]. However, the actual numbers of people infected are estimated to be 20–40 times as many as the diagnosed cases since SINV is neither a well-known or notifiable disease [18], thus human data is highly unreliable. A striking attribute of 2009 was that an unexpected number of *Cx*. *torrentium* and *Cx*. *pipiens* mosquitoes were collected [26]. To present knowledge, these species are not known to bite humans in Sweden, however detailed blood-meal analysis studies on field caught specimens have not yet been performed. The bridge-vector *Ae*. *cinerus* and the potential bridge-vector *Aedes rossicus* were also plentiful in 2009 (J.O. Lundström and J.C. Hesson unpublished data). Thus, although SINV transmission was extraordinarily intense among birds and enzootic vector mosquitoes and mosquito abundance was high, it did not result in an increased number of reported cases of human disease in 2009.

A difficulty in temporal seroprevalence studies can be to determine when the initial infection was acquired. The duration of immunity in birds varies between a few weeks to years depending on bird species as well as virus type and initial viremia [32–34]. Experimentally infected Swedish Passeriformes started producing neutralizing antibodies later than five days post inoculation with SINV, and reached 73% positive individuals after one month. After three months only 15% of the infected Passeriformes had detectable antibodies and one year after infection no bird retained detectable antibodies. For comparison, 71% of Swedish Anseriformes produced detectable antibodies already after five days, and these were still detectable one year after infection in 42% of tested birds [21]. Thus the antibody prevalence detected in Passeriformes in this study represents infections that were acquired five days to three months before sampling, i.e. in the current season or at the overwintering grounds for birds sampled in early summer [21]. It cannot be excluded that after hatching year birds without detectable antibodies have been infected in earlier seasons and, despite the absence of detectable antibodies, are still immune to SINV. Such long lasting protection have been shown for St Louis encephalitis virus (SLEV) where birds are immune to re-infection despite that antibodies cannot longer be detected [33–35].

Birds that are hatched within the study region and during the year of sampling provide the most solid data on new and locally acquired infections. For newly hatched birds, it is estimated that about 20% can be protected by maternal antibodies for up to nine days, i.e. the first period when they are sparsely feathered and bound to the nest, after which they are susceptible to infection [36]. Our 67 positive hatchlings were all sampled after they had left the nests and should thus be free of maternal antibodies and locally infected in the specific summer. Likewise, the detection of SINV antibodies in ten captive birds, born and bred at a local site in Sweden, and in two of the ten weeks old and two of the six weeks old Canada geese (*Branta canadensis*) in mid July 1988 [23], and hatching year European robin, Song thrush, Yellowhammer and Redwing [20] are also evidence of local transmission. We found that there was an increase in SINV antibody prevalence in hatchlings from early to late summer in line with the results for all birds in this study (Table 2) and earlier findings by Lundström et al. [20]. The first virus isolates in mosquitoes are from the middle of July, despite massive virus screening effort of potential vector mosquitoes also earlier in the summer, with 33% of the total season catch of *Ae*. *cinereus* (n = 10780) and 65% of the total season catch of *Cx*. *pipiens/torrentium* (n = 1267) [19]. The early summer findings of newly infected hatchlings and the increasing prevalence of SINV antibodies in the bird population towards late summer show that there is local transmission in the bird population and a subsequent build up of infections before the virus can be detected in the mosquitoes, in agreement with what has been indicated in earlier studies [19,20].

Birds that have detectable antibodies very early in the season could have been infected in their overwintering grounds, or possibly have been bitten by an overwintering female *Cx*. *torrentium* or *Cx*. *pipiens* taking its first blood meal after hibernation. In the present study the earliest hatchlings with SINV antibodies were a European robin on June 21^st^, a Great tit on June 24^th^ and a Song thrush on June 28^th^. The earliest previous detection of antibodies in a hatching year bird is from a redwing on June 15^th^, 1990 [20]. The very earliest findings of a SINV positive bird in the present study is from May 13^th^, and in total 58 non-hatching year birds were found positive in early summer. *Culex* females exit their winter hideouts in March/April and will blood feed before they lay their eggs. These eggs produce the next generation of *Culex* that are ready to emerge as adults in late June. A locally acquired infection before end of June would indicate vertical transmission in mosquitoes, since the last female generation of *Cx*. *torrentium* and *Cx*. *pipens* in the previous summer do not take a blood meal before entering their overwintering grounds. However, empiric data on SINV occurrence in early season mosquitoes is lacking, and it still remains unknown how the local enzootic transmission of SINV is started every season.

Bird-associated viruses can potentially be transferred between distant geographical locations by migrating birds. SINV has a large geographical distribution in the old world and isolates from Sweden show most resemblance to isolates from South Africa, and have less in common with isolates from central and southern Europe [37]. For Western equine encephalomyelitis virus (WEEV) and West Nile virus (WNV) it has been shown that virus can persist in a bird as a chronic latent infection [38,39]. Mosquito vectors could thus theoretically be infected after a recurrent viremia induced by migratory restlessness, such as the reactivation of *Borrelia garinii* infection in Redwings subjected to stress [40]. Such transfer of virus could potentially be undertaken by long distance migrants, migrating between Sweden and South Africa. There are two such species tested in this study; the Willow warbler had antibodies in 2009, with the earliest detected May 20^th^, and the European pied flycatcher, with no antibodies found. Directed sampling of 328 migrating birds arriving in Sweden was performed in spring 1983 and no SINV antibodies were found, while SINV antibody positive birds (10/136) was detected in July and August the same year [19]. Similarly, serology on migrating birds arriving in Finland during spring did not reveal any samples with SINV antibodies [6]. Thus, although reactivation of viremia is theoretically possible, import of virus with migrating birds is likely very rare.

This study confirmed that Turdidae species, especially the *Turdus* species, are often infected with SINV, and further proved that these birds show significant differences in antibody prevalence between years as well as a significant within-year seasonal increase. Thus Turdidae species appears to play the most important role for fluctuations in SINV transmission, and specifically Fieldfare, Redwing and Song thrush would be useful as local sentinels for surveillance of SINV transmission. For most other mosquito-borne bird viruses in the world, such as WEEV and Eastern equine encephalomyelitis virus (EEEV) (*Alphavirus*), SLEV and WNV (*Flavivirus*), Passeriform species are considered the most important hosts, often specifically involving Turdidae species [41–45]. A key factor making Passeriformes suitable hosts is their high abundance close to human settings. In Sweden, more than 90% of the breeding bird population are Passeriformes, and Fieldfare, Redwing, Song thrush and Chaffinch comprise 18% of the Swedish bird fauna [46]. A comparison of bird captures in central and southern Sweden showed that these species were more common in central Sweden, which coincides with human disease that occurs in central but not southern Sweden [18,20], indicating the importance of population density of the most important hosts. Detailed transect studies on densities of thrushes would be an interesting way to get more detailed information on the host abundance. Similarly, detailed studies on the antibody prevalence in Passeriforme birds in Finland would be of high interest since here Galliform species (Capercaillie (*Tetrao urogallus*) and Black Grouse (*Tetrao tetrix*)) are often suggested as main hosts of SINV, based on two human outbreaks that have coincided with population crashes of Grouse [5,13]. Four Galliform species have been investigated in the Swedish studies, and antibodies have been found in 17% (n = 24) of the investigated Capercaillie [23]. Four Capercaillie individuals have also been experimentally infected with SINV and all reached viremias >6.4 PFU/ml [22]. This shows that Cappercaillie is a competent host of SINV and that it is infected in nature. However, Cappercaillies are rare in Sweden, and their habitat is not in close proximity to humans. Thus, their role in SINV transmission in Sweden is of less importance than the passerine birds of the Turdidae family.

In conclusion, this study found no cyclic variation of SINV infections in the bird host population in the River Dalälven floodplains in Sweden. The causes of the yearly variations in the prevalence of SINV neutralizing antibodies in birds and its potential impact on the number of human cases of disease remain to be uncovered.

## References

[1] Rossow H, Ollgren J, Hytonen J, Rissanen H, Huitu O, Henttonen H, et al Incidence and seroprevalence of tularaemia in Finland, 1995 to 2013: regional epidemics with cyclic pattern. Euro Surveill 2015;20: 21209 26314404 10.2807/1560-7917.es2015.20.33.21209

[2] Kallio ER, Begon M, Henttonen H, Koskela E, Mappes T, Vaheri A, et al Cyclic hantavirus epidemics in humans predicted by rodent host dynamics. Epidemics 2009;1: 101–107. 10.1016/j.epidem.2009.03.002 21352757

[3] Niklasson B, Espmark Å, Lundström J. Occurrence of arthralgia and specific IgM antibodies three to four years after Ockelbo disease. J Inf Dis 1988;157: 832–5. 2831289 10.1093/infdis/157.4.832

[4] Kurkela S, Helve T, Vaheri A, Vapalahti O. Arthritis and arthralgia three years after Sindbis virus infection: Clinical follow-up of a cohort of 49 patients. Scand J Infect Dis 2008;40: 167–173. 17852949 10.1080/00365540701586996

[5] Brummer-Korvenkontio M, Vapalahti O, Kuusisto P, Saikku P, Manni T, Koskela P, et al Epidemiology of Sindbis virus infections in Finland 1981–96: possible factors explaining a peculiar disease pattern. Epidemiol Infect 2002;129: 335–345. 12403109 10.1017/s0950268802007409 PMC2869892

[6] Kurkela S, Rätti O, Huhtamo E, Uzcátegui NY, Nuorti JP, Laakkonen J, et al Sindbis virus infection in resident birds, migratory birds, and humans, Finland. Emerg Infect Dis 2008;14: 41–47. 10.3201/eid1401.070510 18258075 PMC2600146

[7] Kurkela S, Manni T, Myllynen J, Vaheri A, Vapalahti O. Clinical and laboratory manifestations of Sindbis virus infection: Prospective study, Finland, 2002–2003. J Inf Dis 2005;191: 1820–1829. 15871114 10.1086/430007

[8] Kurkela S, Manni T, Vaheri A, Vapalathi O. Causative agent of Pogosta disease isolated from blood and skin lesions. Emerg Infect Dis 2004;10: 889–94. 15200824 10.3201/eid1005.030689 PMC3323234

[9] Sane J, Kurkela S, Levanov L, Nikkari S, Vaheri A, Vapalahti O. Development and evaluation of a real-time RT-PCR assay for Sindbis virus detection. J Virol Methods 2012;179: 185–188. 10.1016/j.jviromet.2011.10.021 22079621

[10] Sane J, Kurkela S, Putkuri N, Huhtamo E, Vaheri A, Vapalahti O. Complete coding sequence and molecular epidemiological analysis of Sindbis virus isolates from mosquitoes and humans, Finland. J General Virol 2012;93: 1984–90. 10.1099/vir.0.042853-0 22647374

[11] Sane J, Kurkela S, Desdouits M, Kalimo H, Mazalrey S, Lokki ML, et al Prolonged myalgia in Sindbis infection: Case description and in vitro infection of myotubes and myoblasts. J Inf Dis 2012;206: 407–414. 22615321 10.1093/infdis/jis358

[12] Sane J, Guedes S, Ollgren J, Kurkela S, Klements P, Vapalahti O, et al Epidemic Sindbis virus infection in Finland: Populations-based case-control study of risk factors. J Inf Dis 2011;204: 459–466. 21742846 10.1093/infdis/jir267

[13] Sane J, Guedes S, Kurkela S, Lyytikäinen O, Vapalahti O. Epidemiological analysis of mosquito-borne Pogosta disease in Finland, 2009. Euro Surveill 2010;15: 19462 20085692 10.2807/ese.15.02.19462-en

[14] Jalava K, Sane J, Ollgren J, Ruuhela R, Ratti O, Kurkela S, et al Climatic, ecological and socioeconomic factors as predictors of Sindbis virus infections in Finland. Epidemiol Infect 2013;141: 1857–1866. 10.1017/S095026881200249X 23158410 PMC9155282

[15] Manni T, Kurkela S, Vaheri A, Vapalahti O. Diagnostics of Pogosta disease: Antigenic properties and evaluation of Sindbis virus IgM and IgG enzyme immunoassays. Vector Borne Zoonotic Dis 2008;8: 303–311. 10.1089/vbz.2007.0623 18380591

[16] The Public Health Agency of Sweden. 2015. Stockholm. Sweden. Available: www.folkhalsomyndigheten.se

[17] Bergqvist J, Forsman O, Larsson P, Naslund J, Lilja T, Engdahl C, et al Detection and isolation of sindbis virus from mosquitoes captured during an outbreak in Sweden, 2013.Vector Borne Zoonotic Dis 2015;15: 133–140. 10.1089/vbz.2014.1717 25700044

[18] Lundström JO, Vene S, Espmark Å, Engvall M, Niklasson B. Geographical and temporal distribution of Ockelbo disease in Sweden. Epidemiol Infect 1991;106: 567–574. 1646735 10.1017/s0950268800067637 PMC2271872

[19] Francy DB, Jaenson TG, Lundström JO, Schildt EB, Espmark Å, Henriksson B, et al Ecologic studies of mosquitoes and birds as hosts of Ockelbo virus in Sweden and isolation of Inkoo and Batai viruses from mosquitoes. Am J Trop Med Hyg 1989;41: 355–363. 2572178

[20] Lundström JO, Lindström K, Olsen B, Dufva R, Krakower DS. Prevalence of Sindbis virus neutralizing antibodies among Swedish passerines indicates that thrushes are the main amplifying hosts. J Med Entomol 2001;38: 289–297. 11296837 10.1603/0022-2585-38.2.289

[21] Lundström JO, Niklasson B. Ockelbo virus (Togaviridae: Alphavirus) neutralizing antibodies in experimentally infected Swedish birds. J Wildl Dis 1996;32: 87–93. 10.7589/0090-3558-32.1.87 8627942

[22] Lundström JO, Turell MJ, Niklasson B. Viremia in three orders of birds Anseriformes, Galliformes and Passeriformes) inoculated with Ockelbo virus. J Wildl Dis 1993;29: 189–195. 8387608 10.7589/0090-3558-29.2.189

[23] Lundström JO, Turell MJ, Niklasson B. Antibodies to Ockelbo virus in three orders of birds (Anseriformes, Galliformes and Passeriformes) in Sweden. J Wildl Dis 1992;28: 144–147. 1312646 10.7589/0090-3558-28.1.144

[24] Lundström JO, Niklasson B, Francy DB. Swedish *Culex torrentium* and *Cx*. *pipiens* (Diptera:Culicidae) as experimental vectors of Ockelbo virus. J Med Entomol 1990;27: 561–563. 2167372 10.1093/jmedent/27.4.561

[25] Turell MJ, Lundström JO, Niklasson B. Transmission of Ockelbo virus by *Aedes cinereus*, *Ae*. *communis* and *Ae*. *excrucians* (Diptera: Culicidae) collected in an enzootic area in central Sweden. J Med Entomol 1990;27:266–268. 2159073 10.1093/jmedent/27.3.266

[26] Hesson JC, Verner-Carlsson J, Larsson A, Ahmed R, Lundkvist Å, Lundström JO. *Culex torrentium* mosquito role as major enzootic vector defined by rate of Sindbis virus infection, Sweden, 2009. Emerg Infect Dis 2015;21: 875–878. 10.3201/eid2105.141577 25898013 PMC4412225

[27] Svensson L. Identification guide to European passerines. 4th ed. Stockholm, Sweden; 1992.

[28] Museum of Natural History, Stockholm, Sweden.

[29] R Core Team. R: A language and environment for statistical computing R Foundation for Statistical Computing Vienna, Austria; 2012

[30] Fox J, Weisberg S. An R Companion to Applied Regression. 2nd ed. California: Sage Publications, Thousand Oaks; 2011.

[31] McIntosh BM, Jupp PG, Dos Santos I, Meenehan GM. Epidemics of West Nile and Sindbis viruses in South Africa with *Culex (Culex) univittatus* Theobald as vector. S Afr J Sci 1976;72: 295–300.

[32] Nemeth NM, Oesterle PT, Bowen RA. Humoral Immunity to West Nile Virus Is Long-Lasting and Protective in the House Sparrow (*Passer domesticus*). Am J Trop Med Hyg 2009;80: 864–869. 19407139 PMC2693945

[33] Reisen WK, Kramer L, Chiles RE, Green EG, Martinez VM. Encephalitis virus persistence in California birds: preliminary studies with house finches. J Med Entomol 2001;38: 393–399. 11372964 10.1603/0022-2585-38.3.393

[34] McLean RG, Mullenix J, Kerschner J, Hamm J. The house sparrow (*Passer domesticus*) as a sentinel for St. Louis encephalitis virus. Am J Trop Med Hyg 1983;32: 1120–1129. 6312819 10.4269/ajtmh.1983.32.1120

[35] Reisen WK, Chiles RE, Martinez VM, Fang Y, Green EN. Experimental infection of California birds with western equine encephalomyelitis and St. Louis encephalitis viruses. J Med Entomol 2003;40: 968–982. 14765678 10.1603/0022-2585-40.6.968

[36] Nemeth NM, Oesterle PT, Bowen RA. Passive Immunity to West Nile Virus Provides Limited Protection in a Common Passerine Species. Am J Trop Med Hyg 2008;79: 283–290. 18689637

[37] Lundström JO, Pfeffer M. Phylogeographic structure and evolutionary history of Sindbis Virus. Vector Borne Zoonotic Dis 2010;10: 889–907. 10.1089/vbz.2009.0069 20420530

[38] Nemeth N, Young G, Ndaluka C, Bielefeldt-Ohmann H, Komar N, Bowen R. Persistent West Nile virus infection in the house sparrow (*Passer domesticus*). Arch. Virol 2009;154: 783–789. 10.1007/s00705-009-0369-x 19347246

[39] Reeves WC, Hutson GA, Bellamy RE, Scrivani RP. Chronic Latent Infections of Birds with Western Equine Encephalomyelitis Virus. Proc Soc Exp Biol Med 1958;97: 733–736. 13554464 10.3181/00379727-97-23862

[40] Gylfe Å, Bergström S, Lundström J, Olsen B. Reactivation of Borrelia infecton in birds. Nature 2000;403: 724–725 10693792 10.1038/35001663

[41] Komar N, Langevin S, Hinten S, Nemeth N, Edwards E, Hettler D, et al Experimental infection of North American birds with the New York 1999 strain of West Nile virus. Emerg Infect Dis 2003;9: 311–322. 12643825 10.3201/eid0903.020628 PMC2958552

[42] Reisen WK, Fang Y, Martinez VM. Avian host and mosquito (Diptera: Culicidae) vector competence determine the efficiency of West Nile and St. Louis encephalitis virus transmission. J Med Entomol 2005;42: 367–375. 15962789 10.1093/jmedent/42.3.367

[43] Reisen WK, Lundström JO, Scott TW, Eldridge BF, Chiles RE, Cusack R, et al Patterns of avian seroprevalence to Western equine encephalomyelitis and Saint Louis Encephalitis viruses in California, USA. J Med Entomol 2000;37: 507–527. 10916291 10.1603/0022-2585-37.4.507

[44] Molaei G, Huang S, Andreadis TG. Vector-Host Interactions and Epizootiology of Eastern equine encephalitis Virus in Massachusetts. Vector Borne Zoonotic Dis 2013;13: 312–323. 10.1089/vbz.2012.1099 23473221

[45] Molaei G, Andreadis TG, Armstrong PM, Anderson JF, Vossbrinck CR. Host feeding patterns of *Culex* mosquitoes and West Nile virus transmission, northeastern United States. Emerg Infect Dis 2006;12: 468–474. 16704786 10.3201/eid1203.051004 PMC3291451

[46] Ulfstrand S, Högstedt G. How many birds breed in Sweden? Anser 1976;15: 1–32.

